# From empirical hemostasis to precision reversal: clinical challenges, technological innovations, and individualized strategies in anticoagulant-associated bleeding

**DOI:** 10.3389/fcvm.2026.1846208

**Published:** 2026-06-25

**Authors:** Wei Zheng, Yunqiao Wang, Jing Lu, Mengyang Long, Cuiyao Tang, Zihui Xu, Han Li

**Affiliations:** 1 Department of Traditional Chinese Medicine, Second Affiliated Hospital of Army Medical University, Chongqing, China; 2 Department of Special War Wound, Daping Hospital, Army Medical University, Chongqing, China

**Keywords:** anticoagulants, clinical decision support, hemorrhage, point-of-Care systems, precision medicine, prothrombin complex concentrates, reversal agents

## Abstract

**Background:**

Anticoagulant therapy is indispensable for thromboembolism management, yet it presents an inherent pharmacological paradox by elevating the risk of catastrophic hemorrhage. Navigating this dynamic hemostatic equilibrium remains a profound clinical challenge.

**Pharmacological landscape:**

This review systematically evaluates the evolution of procoagulant strategies. While specific reversal agents (e.g., idarucizumab, andexanet alfa) have inaugurated an era of targeted precision reversal through high-affinity molecular neutralization, their widespread application is frequently hampered by exorbitant pharmacoeconomic costs, limited accessibility, and latent prothrombotic “off-target” effects (e.g., TFPI inhibition). Conversely, non-specific agents, epitomized by four-factor prothrombin complex concentrate (4F-PCC) and adjunctive antifibrinolytics, retain a foundational, highly cost-effective role in real-world rescue paradigms.

**Technological innovations:**

To circumvent existing limitations, current technological trajectories are pivoting towards the rational design of broad-spectrum synthetic molecules (e.g., ciraparantag) and highly programmable nucleic acid aptamer systems. These innovations promise to deliver “on-demand” reversibility and democratize access to hemostatic therapies.

**Clinical management & perspectives:**

Translating these pharmacological breakthroughs into optimal patient outcomes necessitates a paradigm shift in clinical workflows. We propose a multidimensional framework driven by point-of-care (POC) viscoelastic testing, algorithmic clinical decision support systems, and sequential therapy pathways managed by multidisciplinary “Code Bleed” teams. Future research must urgently prioritize large-scale, head-to-head randomized trials focused on patient-centered hard endpoints, ultimately refining evidence-based strategies to maximize therapeutic efficacy while strictly mitigating thrombotic risks.

## 1 Introduction

Anticoagulant therapy is indispensable for stroke prevention in atrial fibrillation (AF) and the management of venous thromboembolism (VTE) (1). However, by interfering with the coagulation cascade to reduce thrombotic burden, these agents inevitably compromise physiological hemostasis, thereby elevating bleeding risk (2). This inherent pharmacological paradox constitutes the central dilemma of anticoagulation management.

Current anticoagulants fall into three broad categories. Vitamin K antagonists (VKAs), such as warfarin, inhibit the synthesis of vitamin K-dependent coagulation factors; their clinical utility is constrained by substantial interindividual variability and the requirement for frequent INR monitoring. Direct oral anticoagulants (DOACs)—comprising the direct thrombin inhibitor dabigatran and the factor Xa inhibitors rivaroxaban, apixaban, and edoxaban—target specific components of the coagulation cascade, offering predictable pharmacokinetics without the need for routine coagulation monitoring (3). Parenteral agents, including unfractionated heparin (UFH), low-molecular-weight heparin (LMWH), and fondaparinux, are reserved for initial therapy or scenarios where oral administration is unfeasible (4).

Bleeding risk remains the pervasive concern throughout anticoagulant therapy (Table 1). The International Society on Thrombosis and Haemostasis (ISTH) has standardized bleeding events into major bleeding and clinically relevant non-major bleeding (CRNMB), enabling unified severity assessment and stratified management. Bleeding profiles differ markedly across anticoagulant classes: warfarin carries a relatively high risk of intracranial hemorrhage (ICH), closely linked to INR stability, whereas DOACs reduce ICH risk by approximately 50% yet increase gastrointestinal (GI) bleeding, particularly with dabigatran and rivaroxaban (5). Therapeutic challenges in special populations—including patients with renal impairment, the frail elderly, and obesity, where altered drug distribution volumes and clearance rates complicate DOAC dosing—further compound management complexity (6). Drug-drug interactions and the clinical paradox arising from the absence of routine DOAC monitoring add additional layers of difficulty.

**Table 1:** Overview of the mechanisms of action and bleeding risk characteristics of mainstream anticoagulants.

Drug category	Representative agents	Key target/mechanism	Monitoring requirement	Characteristic bleeding risk
Traditional oral anticoagulants (VKAs)	Warfarin	Inhibits synthesis of vitamin K-dependent factors (II, VII, IX, X)	Routine INR monitoring mandatory	High inter-individual variability; elevated ICH risk, closely INR-dependent
Direct thrombin inhibitor (DTI)	Dabigatran	Directly inhibits factor IIa (thrombin)	No routine monitoring	≈50% lower ICH vs. warfarin; significantly increased GI bleeding
Factor Xa inhibitor	Rivaroxaban, Apixaban, Edoxaban	Direct inhibition of factor Xa	No routine monitoring	Increased GI bleeding (especially rivaroxaban); lower ICH vs. warfarin
Parenteral anticoagulants	UFH, LMWH, Fondaparinux	ATIII-mediated inhibition of IIa and/or Xa	Drug-dependent (aPTT/anti-Xa)	Insufficient effect → thrombosis; excessive effect → bleeding

*ICH, intracranial hemorrhage; GI, gastrointestinal; VKAs, vitamin K antagonists; INR, international normalized ratio; UFH, unfractionated heparin; LMWH, low-molecular-weight heparin; ATIII, antithrombin III.

**Figure 1:**
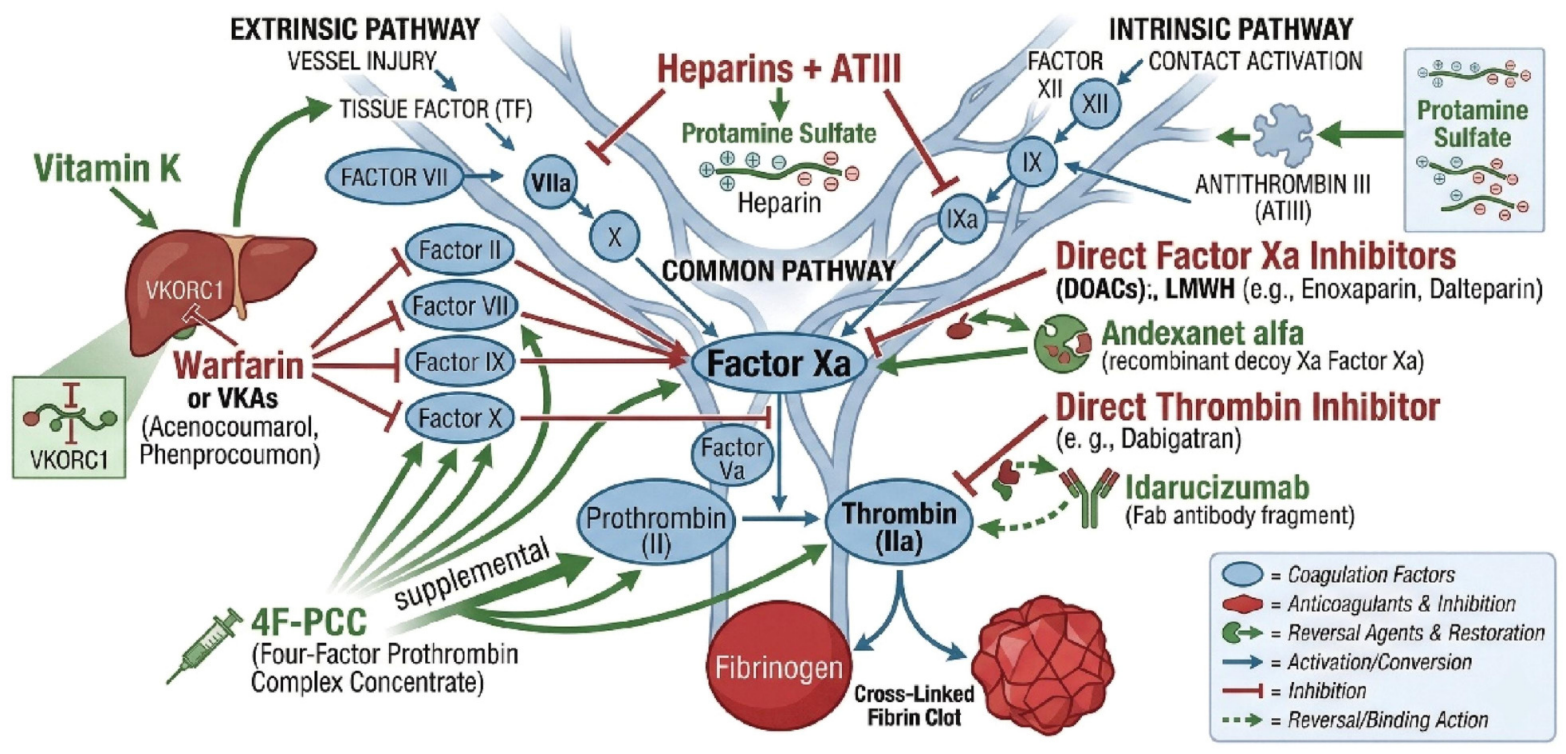
The coagulation cascade and drug/antidote targets.

When anticoagulant-induced bleeding occurs, or when intrinsic coagulopathy substantially elevates hemorrhagic risk, the rational deployment of procoagulant or specific reversal agents becomes a critical component of clinical decision-making (7) (Figure 1).

In the present review, we provide a comprehensive synthesis of current evidence on the pharmacological management of anticoagulant-associated bleeding, encompassing both specific reversal agents and non-specific hemostatic strategies, as well as emerging technologies and clinical decision-making frameworks. This is a narrative review rather than a systematic review. The literature search was conducted in PubMed and Embase for articles published in English from January 2000 to December 2025, using combinations of key terms including “anticoagulant reversal,” “procoagulant agents,” “andexanet alfa,” “idarucizumab,” “prothrombin complex concentrate,” “tranexamic acid,” “protamine,” “ciraparantag,” “aptamer anticoagulant reversal,” and “anticoagulant-associated bleeding management.” Reference lists of retrieved articles and relevant clinical practice guidelines were also manually screened for additional sources. Given the narrative nature of this review, no formal quality assessment or quantitative synthesis was performed; rather, we aimed to provide a clinically oriented, critical appraisal of the evolving therapeutic landscape.

## 2 The hemostatic armamentarium: mechanism-driven pharmacology and clinical positioning

When anticoagulant-induced hemorrhage occurs, clinicians must navigate a diverse arsenal of hemostatic agents with distinct mechanisms, specificities, and safety profiles. These agents can be broadly divided into specific reversal agents—designed to neutralize individual anticoagulants through high-affinity molecular interactions—and non-specific hemostatic agents, which restore hemostasis through broader, pleiotropic mechanisms. The following sections examine each category in turn, with an emphasis on the practical indications, contraindications, and risk-benefit assessments that inform bedside decision-making.

### 2.1 General principles of procoagulant use: indications, contraindications, and risk-benefit assessment

Procoagulant and hemostatic agents are pharmacological therapies that arrest bleeding by accelerating the coagulation cascade or reducing capillary permeability (8). They are indicated for hemorrhagic disorders driven by coagulation factor deficiencies, platelet abnormalities, or hyperfibrinolysis. Within anticoagulant therapy, their clinical positioning encompasses three principal dimensions. First, they serve as targeted interventions for reversing excessive anticoagulation in the setting of acute hemorrhage. Second, they act as prophylactic hemostatic measures in patients with clear anticoagulation indications who nonetheless carry elevated bleeding risk, thereby maintaining a dynamic equilibrium between thrombotic and hemorrhagic tendencies (9). Third, they provide bridging reversal prior to emergent surgery or invasive procedures, mitigating the risk of iatrogenic hemorrhage while enabling necessary interventions to proceed safely.

The application of procoagulant agents demands strict adherence to a rigorous risk-benefit framework (10). In patients requiring long-term anticoagulation, inappropriate procoagulant administration can negate established antithrombotic benefits and, critically, precipitate iatrogenic thrombotic events. Consequently, clinical deployment requires highly individualized decision-making aimed at accurately identifying the precise balance between hemostatic needs and thrombotic risks—a logic that mirrors the fundamental objectives of anticoagulant therapy itself.

### 2.2 Targeted specific reversal agents: the rise and fall of a paradigm

Specific reversal agents are designed on the principle of precision targeting: by binding with high affinity directly to an anticoagulant molecule or its target, they sterically block its interaction with endogenous coagulation factors, thereby restoring physiological hemostasis without broadly activating the coagulation cascade. This targeted approach promised an era of elegant pharmacological solutions to anticoagulant-associated bleeding. However, as the contrasting fates of andexanet alfa and idarucizumab illustrate, molecular precision alone does not guarantee clinical safety, nor does surrogate laboratory reversal reliably translate into improved patient outcomes.

#### 2.2.1 Anti-factor Xa decoys: the cautionary tale of andexanet alfa

**Figure 2:**
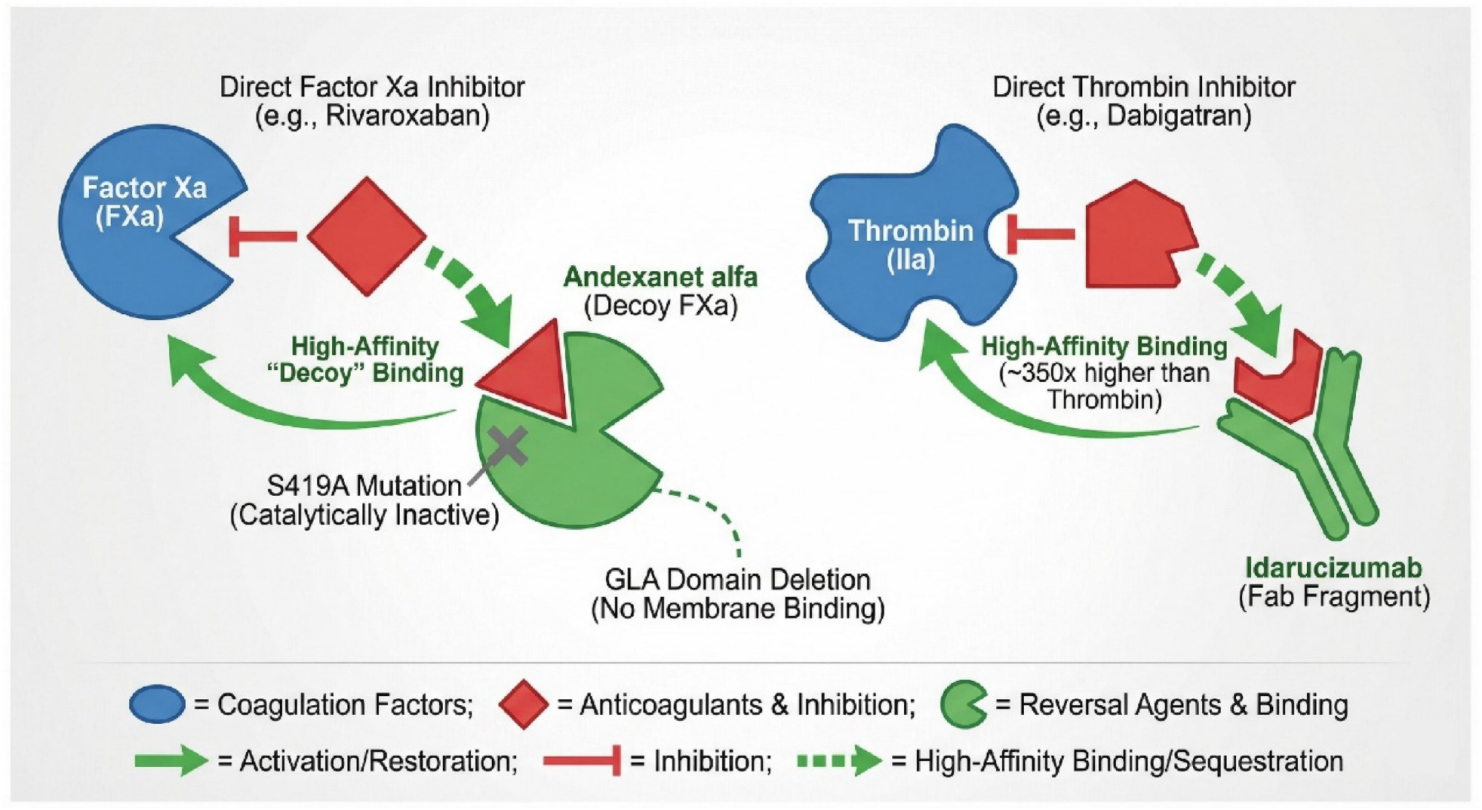
The mechanism of factor Xa/direct thrombin inhibitor antagonists.

Andexanet alfa (11) represented a landmark in targeted anticoagulant reversal: a genetically engineered, recombinant modified human factor Xa decoy protein designed to neutralize factor Xa inhibitors with high specificity. Its structural design (Figure 2) exemplified the principles of precision pharmacology through two critical site-directed mutations (12). First, a mutation at the serine active site rendered it catalytically inert. Second, a mutation within the membrane-binding *γ*-carboxyglutamic acid (Gla) domain prevented its incorporation into the prothrombinase complex. These modifications abolished its capacity to participate in the endogenous coagulation cascade while fully preserving the high-affinity binding pocket for factor Xa inhibitors, creating a decoy receptor that competitively sequestered circulating inhibitors—including rivaroxaban, apixaban, and edoxaban—and restored thrombin generation (13). Intravenous administration rapidly diminished anti-factor Xa activity within minutes, although its effective half-life was notably short at approximately one hour, necessitating a continuous infusion following the initial bolus (14).

The clinical promise of andexanet alfa was initially supported by the ANNEXA-4 trial, a pivotal Phase III multicenter study demonstrating a 92% reduction in median anti-factor Xa activity and an 82% rate of good or excellent hemostatic efficacy at 12 h in patients with factor Xa inhibitor-associated major bleeding (15, 16). These findings provided the evidentiary foundation for regulatory approval. However, the subsequent ANNEXA-I randomized controlled trial—designed to evaluate clinical superiority over usual care—ultimately reversed the narrative. An interim analysis revealed that although andexanet alfa was superior in controlling hematoma expansion in patients with intracranial hemorrhage, it was associated with a significantly higher 30-day incidence of thrombotic events (10% vs. 5.6% in the control group), with no confirmed improvement in long-term functional outcomes or all-cause mortality (16, 17).

This thrombotic signal was not incidental but mechanistically rooted. Beyond neutralizing factor Xa inhibitors, andexanet alfa binds with high affinity to tissue factor pathway inhibitor (TFPI), a critical endogenous negative regulator of the extrinsic coagulation pathway. Inhibition of TFPI removes a key brake on the tissue factor–factor VIIa (TF-FVIIa) complex, triggering unregulated thrombin generation—a so-called “thrombin storm”—that mediates a systemic prothrombotic state. This off-target physiological effect, inherent to the decoy molecule's structure, ultimately led to the agent's withdrawal from the market.

The case of andexanet alfa thus serves as a definitive cautionary tale. It demonstrates that exquisite molecular targeting does not guarantee clinical safety, and that surrogate laboratory endpoints (anti-Xa activity reduction) do not reliably translate into patient-centered outcomes. Its legacy underscores the imperative of rigorous safety evaluation against hard clinical endpoints before targeted reversal agents assume a routine place in clinical care.

#### 2.2.2 Anti-thrombin antibodies: the sustained success of idarucizumab

Dabigatran, a direct thrombin inhibitor, is among the most widely prescribed DOACs (18), valued for its predictable pharmacokinetics, lower ICH risk relative to warfarin, and lack of routine coagulation monitoring. Multiple international guidelines recommend it as first-line therapy for stroke prevention in non-valvular atrial fibrillation and for VTE management (19, 20). In the settings of life-threatening major bleeding or urgent invasive procedures, rapid reversal of its anticoagulant effect remains a critical clinical necessity (21).

Idarucizumab (22) is a humanized monoclonal antibody fragment (Fab) specifically engineered to bind dabigatran with high affinity—approximately 350 times greater than dabigatran's affinity for thrombin—and essentially irreversible specificity (Figure 2). Through competitive displacement (23), it rapidly uncouples dabigatran from the active site of thrombin, forming a stable, biologically inactive complex that reinstates the physiological coagulation cascade. Idarucizumab exhibits no cross-reactivity with endogenous thrombin, other coagulation factors, or non-targeted anticoagulants (24). Critically, the antibody fragment is devoid of intrinsic procoagulant activity (25), meaning it does not independently trigger clot formation.

The landmark RE-VERSE AD trial—a global, multicenter, single-arm Phase III study enrolling 503 dabigatran-treated patients (301 with life-threatening major bleeding; 202 requiring urgent procedures)—demonstrated complete anticoagulant reversal within minutes of a 5 g intravenous infusion (26, 27). Hemostasis was achieved in 93% of the urgent surgery cohort. The 30-day thromboembolic event rate was 4.8%, with the vast majority occurring in patients who had not yet reinitiated baseline anticoagulation—underscoring that reversal itself restores the patient's underlying thrombotic predisposition rather than reflecting an intrinsic prothrombotic effect of the antibody. Subsequent neurosurgical subgroup analyses (27) and the real-world RE-VECTO registry (*n* = 359) further corroborated its hemostatic efficacy, with only 1.7% requiring a second dose (28). Based on this evidence, guidelines from the ISTH and ACC/AHA unanimously recommend idarucizumab as the first-line reversal strategy for dabigatran-associated life-threatening bleeding and for pre-procedural reversal (29). It remains the only DOAC-specific reversal agent approved for urgent surgical interventions in non-bleeding patients (30).

Clinically, idarucizumab is administered as a fixed 5 g intravenous dose, delivered as two consecutive 2.5 g boluses no more than 15 min apart (27, 31). Dose adjustment is unwarranted for age, hepatic, or renal function (32); although renal impairment may prolong the terminal half-life, reversal efficacy remains uncompromised (33). Given its exquisite target specificity, idarucizumab neutralizes only dabigatran and has no reversal effect on factor Xa inhibitors or VKAs; thus, definitive confirmation of dabigatran use is a prerequisite (34). For laboratory monitoring, ECT and dTT serve as the recommended quantitative assays, while aPTT provides only a qualitative, supplementary indicator (35).

#### 2.2.3 Classical reversal agents: protamine and vitamin K

Vitamin K: Vitamin K serves as the specific antidote for warfarin (36, 37), which exerts its anticoagulant effect by inhibiting vitamin K epoxide reductase (VKOR), thereby blocking the recycling of vitamin K and impairing the *γ*-carboxylation of vitamin K-dependent coagulation factors (II, VII, IX, X) (38, 39). Exogenous vitamin K restores hepatic synthesis of functional coagulation factors, reversing the anticoagulant effect at its molecular source (40). Onset of action is route-dependent: intravenous infusion initiates reversal within 4–6 h (41), whereas oral administration is delayed to approximately 24 h, with complete reversal often requiring several days (42). Clinically, vitamin K is indicated for patients with supratherapeutic INRs and significant bleeding. For life-threatening major hemorrhage, international guidelines recommend co-administration with 4F-PCC to achieve both rapid hemostasis and sustained reversal.

Protamine Sulfate: Protamine sulfate (43) is the specific antagonist for heparin-based anticoagulants (44). A strongly basic cationic polypeptide originally isolated from salmon sperm (45), protamine neutralizes heparin within minutes through electrostatic binding to highly acidic heparin molecules, forming stable, biologically inactive complexes. Heparins function by binding to antithrombin III (ATIII), accelerating its inhibition of thrombin and factor Xa (46, 47). Protamine's reversal efficacy is molecular-weight-dependent: it achieves approximately 100% neutralization of UFH, yet only approximately 60% neutralization of LMWH (48, 49), owing to the shorter polysaccharide chains of LMWH that offer fewer protamine-binding sites and preferential anti-Xa activity (50).

Critically, protamine itself exhibits dose-dependent anticoagulant activity in the absence of heparin. Therefore, its administration must be predicated upon clear documentation of recent heparin exposure and the time of the last dose. Additional safety concerns include hypersensitivity reactions—patients with fish allergy, prior protamine exposure, or prior use of protamine-containing insulin (e.g., NPH insulin) are at elevated risk (51)—and severe cardiovascular complications (hypotension, bradycardia) associated with rapid intravenous infusion (52). In summary, vitamin K provides slow-onset but sustained reversal targeting upstream factor synthesis, ideally in combination with PCC for major warfarin-associated bleeding; protamine offers rapid, chemical neutralization of UFH and partial reversal of LMWH, but demands rigorous patient selection given its paradoxical anticoagulant potential in the absence of circulating heparin (Table 2).

**Table 2:** Comprehensive information comparison of specific reversal agents.

Agent	Targeted anticoagulant(s)	Mechanism	Dosage regimen	Key Advantages	Limitations/safety risks
Idarucizumab	Dabigatran (DTI)	Humanized Fab fragment; high-affinity, essentially irreversible binding to dabigatran, competitively blocking thrombin inhibition	Fixed 5 g IV, as two 2.5 g boluses ≤15 min apart	Rapid onset; no intrinsic procoagulant activity; no dose adjustment for hepatic or renal impairment	Effective only against dabigatran; no reversal of other anticoagulant classes
Vitamin K	Warfarin (VKA)	Restores hepatic synthesis of functional vitamin K-dependent factors (II, VII, IX, X)	IV or oral; dose titrated to INR	Prolonged duration of action; extremely low cost; flexible administration routes	Slow onset (4–6 h IV; ∼24 h oral); cannot be used as monotherapy for life-threatening major bleeding
Protamine Sulfate	UFH; LMWH (partial)	Strongly basic polypeptide; neutralizes acidic heparin molecules via electrostatic binding, blocking ATIII-mediated anticoagulation	IV bolus; dose matched 1:1 to total heparin administered	Ultra-rapid onset (within 5 min); 100% neutralization of UFH	Only ∼60% neutralization of LMWH; no activity against fondaparinux; risk of hypersensitivity reactions; paradoxical anticoagulant effect in the absence of circulating heparin—administration requires confirmed recent heparin exposure and last dose timing

Note on Andexanet Alfa: Andexanet alfa, a modified recombinant factor Xa decoy receptor, was previously approved for reversal of direct factor Xa inhibitors (rivaroxaban, apixaban). However, the ANNEXA-I trial demonstrated a significantly increased 30-day thrombotic event rate (10% vs. 5.6%) attributed in part to off-target TFPI inhibition, and the agent has subsequently been withdrawn from the market. It is therefore excluded from the current table of clinically available agents. See Section 2.2.1 for a detailed discussion of its mechanism and the safety concerns that led to its withdrawal.

### 2.3 Non-specific hemostatic agents: the reigning workhorses

Despite the conceptual appeal of targeted reversal, practical constraints—limited availability, prohibitive cost, and, in the case of andexanet alfa, unacceptable thrombotic risk—have relegated specific agents to a narrow set of clinical scenarios. In the majority of anticoagulant-associated bleeding emergencies, non-specific hemostatic agents remain the cornerstone of management. These agents restore hemostasis through broad mechanisms: replenishing coagulation substrates, inhibiting fibrinolysis, or stabilizing vascular integrity. The following sections examine the hierarchy of non-specific options, emphasizing the evidence, indications, and safety considerations that guide their clinical use.

#### 2.3.1 Factor concentrates and plasma: a hierarchy of choice

Coagulation factor concentrates are the cornerstone of non-specific hemostatic therapy for anticoagulant-associated major bleeding, particularly when specific reversal agents are unavailable or the offending anticoagulant is unidentified (53, 54). The three principal formulations—4F-PCC, aPCC, and FFP—differ markedly in composition, efficacy, and safety (55), mandating a clear hierarchy of clinical selection.

**Four-Factor Prothrombin Complex Concentrate (4F-PCC)** is the most widely utilized standardized factor concentrate (56). Comprising vitamin K-dependent factors (II, VII, IX, X) balanced with the physiological anticoagulants protein C and protein S (57), 4F-PCC bypasses the coagulation steps blocked by anticoagulants, directly restoring thrombin generation (58). In factor Xa inhibitor-associated major bleeding, Observational data suggest hemostatic efficacy rates of 65%–89% for 4F-PCC in factor Xa inhibitor-associated major bleeding, though these estimates derive from small, single-arm studies and high-quality randomized evidence is lacking. The reported 30-day thromboembolic event rate is 3%–8% (59, 60). Its therapeutic cost is approximately one-quarter to one-fifth that of andexanet alfa (61). An important safety consideration: certain 4F-PCC formulations contain trace amounts of heparin and are therefore contraindicated in patients with confirmed or strongly suspected heparin-induced thrombocytopenia (HIT). 4F-PCC is recommended as the first-line non-specific agent for DOAC-associated major bleeding and for urgent warfarin reversal (in combination with vitamin K).

**Activated Prothrombin Complex Concentrate (aPCC)** contains activated factor VII (FVIIa) in addition to non-activated factors II, IX, and X, directly initiating the common coagulation pathway for a more potent hemostatic effect (62). Its use is strictly reserved for breakthrough bleeding in hemophilia patients with inhibitors (63) or as salvage therapy for DOAC-associated bleeding refractory to 4F-PCC. The thrombotic risk with aPCC is considerably higher than with 4F-PCC, particularly when cumulative doses exceed 100 U/kg/d (64).

**Fresh Frozen Plasma (FFP)** contains the full spectrum of coagulation factors but at low concentrations, necessitating large infusion volumes that predispose patients to transfusion-associated circulatory overload (TACO) and transfusion-related acute lung injury (TRALI) (65, 66). Requirements for ABO compatibility and prolonged thawing further delay correction of coagulopathy relative to 4F-PCC (67). FFP is therefore reserved solely for settings where factor concentrates are unavailable.

#### 2.3.2 Antifibrinolytics: valuable adjuncts, not a solo act

Tranexamic acid (TXA), a synthetic lysine analogue, is the most widely used antifibrinolytic agent, with a robust evidence base supporting its role in traumatic hemorrhage, perioperative bleeding, and as an adjunct in anticoagulant-associated bleeding (68–70). TXA competitively inhibits fibrinolysis by binding to the lysine-binding sites on plasminogen, thereby preventing its conversion to plasmin and stabilizing pre-formed fibrin clots (71). Following intravenous administration, TXA has a half-life of approximately 2 h and provides stable fibrinolytic inhibition for up to 8 h at plasma concentrations of 5–15 mg/L (72, 73). It can be administered intravenously (preferred in acute severe bleeding), orally (for chronic conditions), or topically (for targeted surgical hemostasis) (74, 75).

The clinical efficacy of TXA is firmly established by the CRASH trial program, most notably the CRASH-2 trial (*n* = 20,211), which demonstrated that early administration (within 3 h of injury) of a 1 g intravenous loading dose followed by 1 g over 8 h significantly reduced all-cause mortality in trauma patients without increasing vascular occlusive events (76–78). Subsequent trials have confirmed its hemostatic benefits in traumatic brain injury, postpartum hemorrhage, and major orthopedic surgery (79–81).

In the context of anticoagulant-associated bleeding, TXA serves strictly as an adjunctive agent. It inhibits hyperfibrinolysis but cannot reverse the pharmacological activity of anticoagulants; therefore, it must never be used as standalone monotherapy for major bleeding. Rather, TXA should be co-administered with specific reversal agents (e.g., idarucizumab) or non-specific procoagulants (e.g., 4F-PCC) (82, 83). Ideally, its use should be guided by objective confirmation of hyperfibrinolysis via viscoelastic hemostatic assays (VHAs) (84). Absolute contraindications include active thromboembolic disease and non-traumatic subarachnoid hemorrhage; the hyperfibrinolytic phase of DIC and severe renal impairment constitute relative contraindications (85).

#### 2.3.3 Other adjunctive agents: limited evidence, niche roles

Several additional hemostatic agents occupy niche roles in certain clinical settings, though their use rests on a limited evidence base and they remain absent from mainstream international guideline recommendations.

Hemocoagulase, a thrombin-like enzyme derived from pit viper venom, promotes local platelet adhesion and hydrolyzes fibrinogen to fibrin at bleeding sites (86). Etamsylate is a synthetic agent that reduces capillary endothelial permeability and enhances platelet adhesion, without demonstrable systemic procoagulant activity (87, 88). Plant-derived formulations such as Yunnan Baiyao exert pleiotropic hemostatic effects through local procoagulant activity, fibrinolytic inhibition, and anti-inflammatory tissue repair (89). All three are used predominantly as empirical adjuncts for capillary or superficial bleeding in perioperative or traumatic settings (90, 91).

The evidence supporting these agents consists almost exclusively of small-sample, single-center observational studies; large-scale randomized controlled trials validating their efficacy and long-term safety are lacking (92–94). Their role in anticoagulant-associated major bleeding remains unsubstantiated, and they should be regarded strictly as empirical adjuncts rather than standard-of-care interventions (Table 3).

**Table 3:** Comprehensive information comparison of Non-specific hemostatic agents.

Drug class	Specific agent	Mechanism	Key clinical positioning	Key warnings/contraindications
Coagulation Factor Concentrates	4F-PCC	Replenishes vitamin K-dependent factors (II, VII, IX, X); bypasses inhibited steps to restore thrombin generation	First-line non-specific agent for DOAC-associated major bleeding; preferred agent for urgent warfarin reversal (with vitamin K)	Contraindicated in HIT (some formulations contain trace heparin); active thromboembolic disease; hyperfibrinolytic phase of DIC. Evidence largely observational; no RCT data in DOAC-associated bleeding
	aPCC	Contains activated factor VII (FVIIa); directly initiates common coagulation pathway	Hemophilia A/B with inhibitors; salvage therapy for 4F-PCC-refractory bleeding	Higher thrombotic risk than 4F-PCC, especially >100 U/kg/d; hypercoagulable states; history of thromboembolism
	FFP	Replenishes full spectrum of coagulation factors and plasma proteins	Last-resort option when factor concentrates are unavailable	Volume overload; TRALI; ABO incompatibility; slower correction than 4F-PCC
Antifibrinolytic Agents	Tranexamic Acid (TXA)	Competitively inhibits plasminogen activation; stabilizes fibrin clots	Adjunctive therapy (not monotherapy) for anticoagulant-associated bleeding; first-line for major traumatic hemorrhage	Active thromboembolic disease; non-traumatic subarachnoid hemorrhage; use ideally guided by VHA confirmation of hyperfibrinolysis
Adjunctive Hemostatic Agents	Hemocoagulase	Promotes local platelet adhesion; converts fibrinogen to fibrin	Empirical adjunct for capillary/superficial bleeding	Limited evidence base (small-sample observational studies); not recommended by mainstream guidelines
	Etamsylate	Enhances platelet adhesion; reduces capillary endothelial permeability	Empirical adjunct for minor vascular permeability-related bleeding	Limited evidence base; no high-quality studies in anticoagulant-associated major bleeding
	Yunnan Baiyao	Pleiotropic: local procoagulant, antifibrinolytic, anti-inflammatory	Empirical adjunct for traumatic and perioperative hemostasis	Limited evidence base; undisclosed formulation; not incorporated into international guidelines

## 3 Bridging the gap: emerging innovations to address current limitations

The preceding section underscored the persistent shortcomings of current hemostatic strategies: narrow target specificity, limited accessibility, prohibitive cost, and, most critically, the thrombotic risks that ultimately led to andexanet alfa's withdrawal. These limitations have spurred the development of next-generation technologies designed to circumvent the constraints of traditional antidotes. The innovations now in the pipeline share a common objective: to deliver safer, more accessible, and on-demand reversibility across the full spectrum of anticoagulant therapies. This section surveys three interconnected frontiers: universal reversal agents and programmable aptamer systems, advanced biologics for refractory bleeding, and companion antidotes purpose-built for novel anticoagulants.

### 3.1 The quest for a universal antidote and on-demand reversibility

A central limitation of current targeted reversal agents is their narrow specificity—idarucizumab neutralizes only dabigatran, and no clinically available agent broadly covers factor Xa inhibitors, direct thrombin inhibitors, and heparins alike. This gap has driven the search for universal antidotes capable of binding multiple anticoagulant classes with a single molecular entity.

Ciraparantag (95) is the most advanced candidate in this category. A synthetic, water-soluble, cationic small molecule, ciraparantag binds to diverse anticoagulants—including direct factor Xa inhibitors, direct thrombin inhibitors, UFH, and LMWH—through non-covalent hydrogen bonding and charge-charge interactions, sterically preventing their engagement with endogenous coagulation targets (96, 97). In Phase I and II studies, a single intravenous dose of 100 to 300 mg achieved complete reversal of anticoagulant effects within 10 min, with effects sustained for up to 24 h; the agent showed no intrinsic procoagulant activity, with adverse events limited to transient facial flushing and dysesthesia (98). While these early data are promising, ciraparantag has yet to complete Phase III trials, and its clinical profile—including rare safety signals—remains to be fully defined. It is best regarded as a highly promising investigational agent whose ultimate role awaits rigorous evaluation in definitive outcome studies.

**Figure 3:**
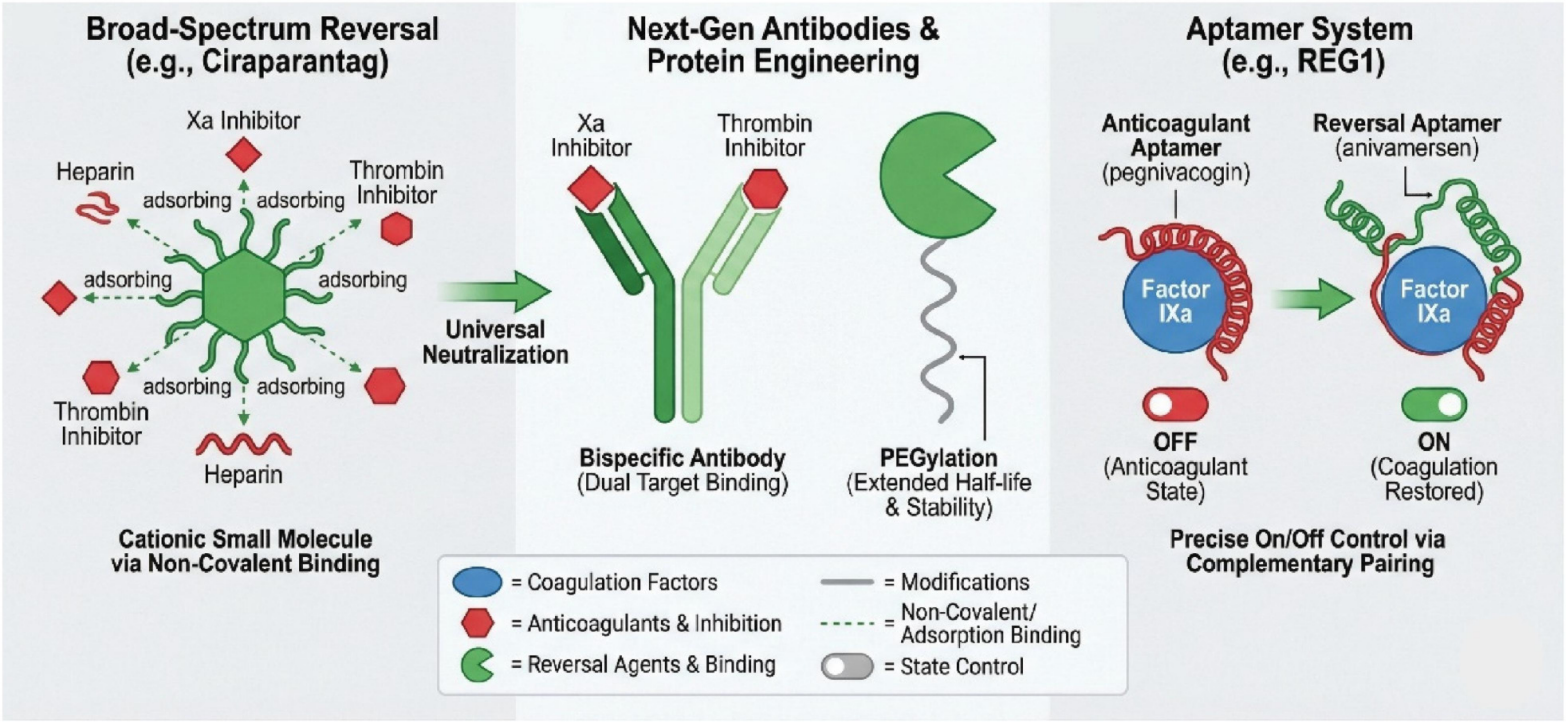
Overview of novel pharmacological entities.

A complementary strategy addresses not only breadth of reversal but also the degree of control over the reversal process itself. Nucleic acid aptamer technology offers a fundamentally different paradigm: paired anticoagulant-reversal systems capable of programmable, “light-switch-like” modulation of the coagulation cascade (99). The REG1 system, comprising pegnivacogin (an RNA aptamer targeting factor IXa) and its complementary reversal oligonucleotide anivamersen, enables clinicians to titrate the degree of anticoagulation reversal by adjusting the dose of the reversal agent (100). This platform is particularly suited to short-term, high-intensity anticoagulation scenarios—such as percutaneous coronary intervention (PCI) and cardiopulmonary bypass—where rapid and precisely controllable reversal is essential (101). Compared with monoclonal antibodies and recombinant proteins, nucleic acid aptamers offer lower synthesis costs, minimal immunogenicity, and high structural programmability, providing a viable pathway toward cost-effective, broadly accessible anticoagulant-reversal systems (102, 103). Ongoing research is focused on optimizing *in vivo* stability and further reducing immunogenicity (Figure 3).

### 3.2 Next-generation recombinant factors and antibody engineering

For the most severe refractory bleeding scenarios—where both specific and non-specific standard agents prove insufficient—advanced biologic therapies continue to evolve.

Recombinant activated factor VII (rFVIIa) bypasses the need for upstream factor activation by binding directly to tissue factor exposed at sites of vascular injury, generating a localized “thrombin burst” on activated platelet surfaces (104, 105). Although formally approved for hemophilia patients with inhibitors, rFVIIa has assumed a vital salvage role in life-threatening anticoagulant-associated bleeding, massive traumatic hemorrhage, and severe coagulopathy of advanced liver disease—contexts where specific reversal agents are either unavailable or inadequate (106). Its use carries an inherent thrombotic risk, and careful patient selection is essential.

A conceptually distinct bypassing strategy is exemplified by VMX-C001 (107), a modified recombinant variant of human factor X (FX) engineered to resist DOAC-mediated inhibition while retaining full procoagulant activity. Unlike andexanet alfa, which sequesters factor Xa inhibitors, VMX-C001 substitutes for endogenous FX that has been functionally silenced, restoring the common coagulation pathway independently of the specific inhibitor used. *In vitro* data demonstrate that VMX-C001 completely normalizes DOAC-prolonged clotting times, in contrast to the partial correction achieved by 4F-PCC. The agent is currently under evaluation for surgical reversal in the Equilibrix trial. If clinically validated, VMX-C001 would occupy a distinct niche as a universal FXa DOAC bypassing agent purpose-built for pre-procedural reversal—a scenario for which no dedicated agent is currently approved.

Building on the success and lessons of idarucizumab, monoclonal antibody technology continues to advance toward bispecific and multispecific constructs capable of neutralizing multiple anticoagulant targets via a single molecular entity (108, 109). Concurrently, protein engineering strategies—including PEGylation and amino acid sequence optimization—aim to extend the half-lives of recombinant reversal agents and reduce the need for continuous infusion, directly addressing a key limitation identified in the case of andexanet alfa (110). These innovations remain predominantly preclinical or in early-phase development.

### 3.3 Companion antidotes for novel anticoagulants

The most consequential lesson of the andexanet alfa experience is that the clinical development of novel anticoagulants must proceed in lockstep with the parallel development of their specific reversal agents. As the pharmacological frontier shifts toward inhibitors of factors upstream in the intrinsic pathway—most notably factor XI (FXI) inhibitors such as milvexian and asundexian, which hold theoretical promise of uncoupling thrombosis prevention from bleeding risk (111)—this lesson assumes heightened urgency. No pharmacological intervention targeting the coagulation cascade can be presumed entirely free of hemorrhagic complications, particularly in the contexts of major trauma or emergency surgery. Accordingly, monoclonal antibodies and small-molecule antagonists targeting FXIa are already under parallel investigation as companion reversal agents (112).

Beyond individual drug-antidote pairs, efforts to improve the accessibility of reversal strategies are equally critical. Research into the refined reconstitution of existing non-specific agents (e.g., targeted modifications of PCCs to approximate the efficacy of specific reversal agents at reduced cost) and the clinical validation of biosimilars and generic small-molecule alternatives such as ciraparantag aim to lower the financial threshold of hemostatic rescue and democratize global access to life-saving interventions (113).

## 4 Refining clinical practice: integrating evidence, technology, and teamwork

While the emerging technologies surveyed in the preceding section hold considerable promise, the clinician confronting an actively bleeding anticoagulated patient today must act on the basis of imperfect evidence, institutional resource constraints, and time-sensitive physiology. Translating pharmacological knowledge into optimal patient outcomes requires not only familiarity with the available agents but also a structured, systems-level approach to decision-making. This section examines three practical dimensions of clinical care: confronting the evidence gaps that complicate agent selection, implementing a pragmatic, risk-stratified management framework, and navigating the secondary decision of when and how to reinitiate anticoagulation after hemostasis is achieved.

### 4.1 Confronting the evidence gap: what limits current decision-making

The evidence base underpinning current reversal strategies carries important limitations that directly affect clinical decision-making.

First, recommendations for specific reversal agents are derived predominantly from single-arm trials or indirect comparisons rather than high-quality, head-to-head randomized controlled trials (114). In factor Xa inhibitor-associated major bleeding, multiple systematic reviews have not established the superiority of andexanet alfa over 4F-PCC in reducing hard clinical endpoints such as all-cause mortality (115). This evidence gap is compounded by the absence of empirical data in populations with distinct pharmacokinetic profiles—patients with end-stage renal disease on hemodialysis, pediatric cohorts, and pregnant women—for whom the safety and efficacy of available agents remain largely uncharacterized (116).

Second, the prohibitive cost of specific reversal agents creates substantial access disparities. When andexanet alfa was on the market, its standard treatment course cost approximately five to ten times that of 4F-PCC (117). In primary care facilities and developing nations, the combination of high stocking costs and low usage frequency means these emergency medications are often unavailable at the point of need, substantially eroding the theoretical advantage of targeted reversal in real-world practice (118).

Third, the thrombotic safety signal that led to andexanet alfa's withdrawal serves as the most tangible illustration of the risk inherent in manipulating the coagulation system. The ANNEXA-I trial demonstrated a significantly higher 30-day thrombotic event rate with andexanet alfa compared with usual care (10% vs. 5.6%) (17), a finding mechanistically attributed to off-target inhibition of tissue factor pathway inhibitor (TFPI), which removes a critical brake on thrombin generation. This episode has fundamentally reshaped the risk-benefit calculus applied to all emerging reversal agents. Fourth, the evidence supporting 4F-PCC—now the de facto standard for factor Xa inhibitor-associated major bleeding—rests predominantly on observational cohorts rather than randomized comparisons, and its hemostatic mechanism in this setting remains incompletely characterized.

### 4.2 A pragmatic algorithm for anticoagulant-associated major hemorrhage

**Figure 4:**
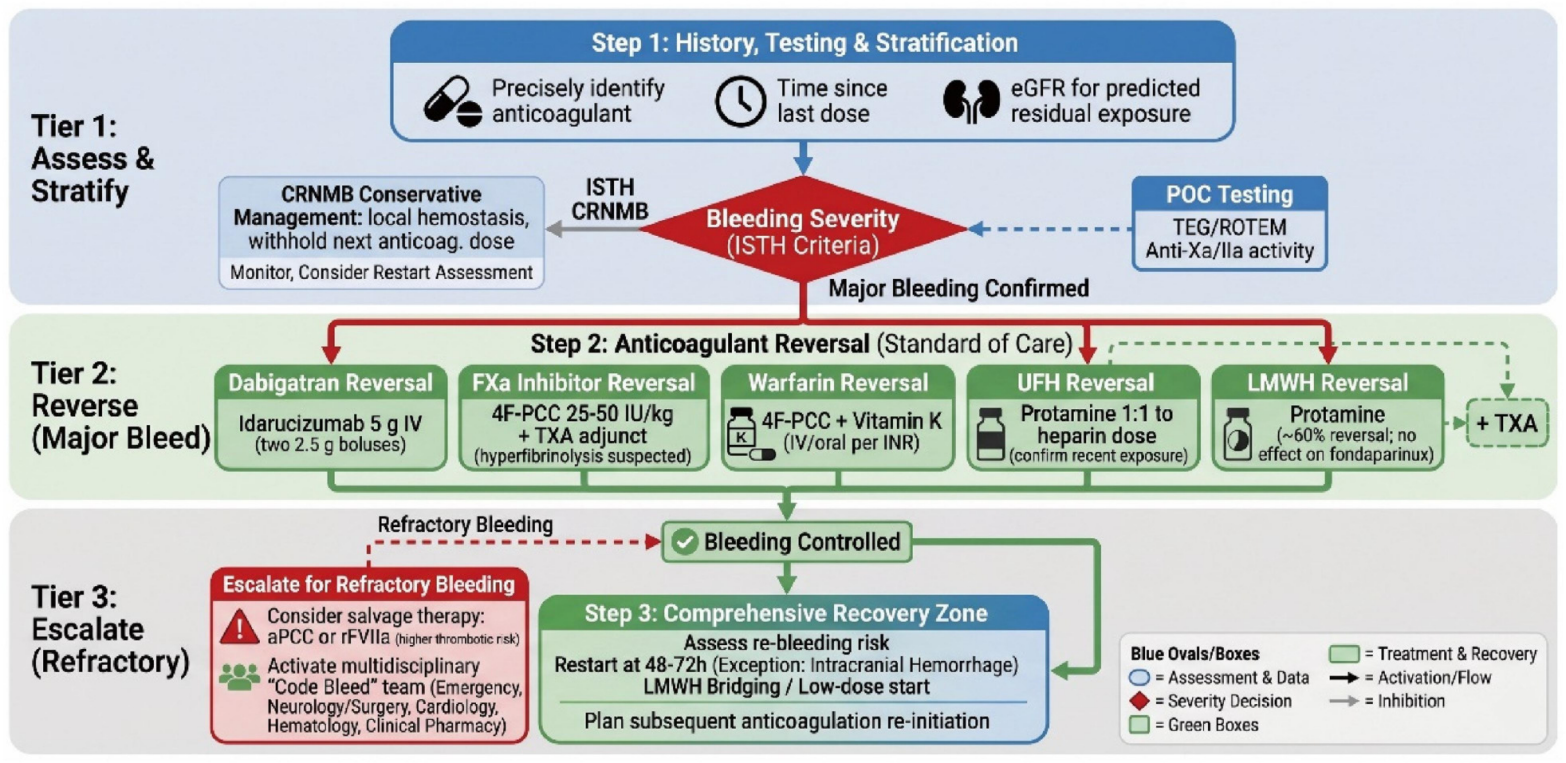
Overview of the “Three-Step” strategy for clinical hemorrhage treatment.

Acknowledging these evidence limitations, clinicians nonetheless require an actionable framework for time-sensitive decision-making. The following algorithm (Figure 4) integrates current guideline recommendations, pharmacological principles, and practical considerations of resource availability into a three-tiered approach.

#### 4.2.1 Tier 1: assessment and stratification

The initial step is to distinguish major bleeding from clinically relevant non-major bleeding (CRNMB) using ISTH criteria. Simultaneously, essential pharmacological history must be obtained: the specific anticoagulant agent, the most recent dose and its timing, and the patient's renal function (eGFR), which critically influences residual drug exposure. Where institutional resources permit, conventional laboratory testing should be supplemented with point-of-care viscoelastic hemostatic assays (VHAs; e.g., TEG/ROTEM) or rapid anti-Xa/IIa activity testing to identify whether the hemorrhage is driven solely by anticoagulant accumulation or is compounded by consumptive coagulopathy and hyperfibrinolysis (119, 120).

For CRNMB, conservative management—local hemostatic measures and temporary withholding of subsequent anticoagulant doses—is generally sufficient and avoids exposing the patient to the thrombotic risks of systemic reversal.

#### 4.2.2 Tier 2: pharmacological reversal — current standard of care

For confirmed major hemorrhage, agent selection is dictated by the identified anticoagulant and institutional drug availability.

Dabigatran-associated major bleeding: Idarucizumab, administered as a fixed 5 g intravenous dose (two 2.5 g boluses), is the guideline-recommended first-line reversal strategy based on the RE-VERSE AD trial data (26, 29).

Factor Xa inhibitor-associated major bleeding: With andexanet alfa no longer available, 4F-PCC is the principal pharmacological option, based on guideline consensus and observational data. Dosed at 25–50 IU/kg depending on institutional protocol, 4F-PCC restores thrombin generation by bypassing the inhibited step in the coagulation cascade (59). Tranexamic acid (TXA) should be co-administered as an adjunct, particularly when hyperfibrinolysis is suspected or confirmed by VHA (84).

Warfarin-associated major bleeding: 4F-PCC, combined with vitamin K, provides rapid hemostasis (via factor replenishment) and sustained reversal (via resumption of endogenous factor synthesis). Vitamin K alone is insufficient for life-threatening hemorrhage due to its slow onset (4–6 h intravenously; ∼24 h orally) (41).

Heparin-associated major bleeding: Protamine sulfate, dosed 1:1 to the total heparin administered, achieves complete UFH neutralization within minutes. For LMWH, reversal is partial (approximately 60%), and protamine has no activity against fondaparinux (48). Prior to administration, clear documentation of recent heparin exposure must be confirmed, given protamine's paradoxical anticoagulant effect in the absence of circulating heparin.

#### 4.2.3 Tier 3: refractory bleeding and multidisciplinary escalation

For bleeding that remains uncontrolled despite Tier 2 interventions, salvage therapies should be considered. Activated prothrombin complex concentrate (aPCC) or recombinant activated factor VII (rFVIIa) may be deployed, with the recognition that both carry a higher thrombotic risk than 4F-PCC (64, 106).

At this stage, activation of a multidisciplinary “Code Bleed” team is critical. This team should integrate emergency medicine, neurology or surgery (for source control), cardiology (for thrombotic risk stratification), hematology and laboratory medicine (for advanced coagulation monitoring), and clinical pharmacy (for pharmacokinetic assessment and dosing guidance) (44, 121).

### 4.3 Re-initiation of anticoagulation: navigating the secondary risk

Once hemorrhage is controlled, the clinician faces the equally consequential decision of when and how to resume anticoagulation—a determination for which definitive consensus guidelines remain lacking (54). Premature re-initiation risks rebleeding; excessive delay exposes the patient to the original thromboembolic risk for which anticoagulation was prescribed.

A cautious, individualized approach is warranted. Once complete hemostatic stability is confirmed—via neuroimaging in cases of intracranial hemorrhage, or comprehensive clinical assessment in extracranial bleeding—baseline anticoagulation should be resumed as early as clinically feasible, typically within 48–72 h post-hemostasis. Intracranial hemorrhage may warrant a significantly longer deferral. To mitigate recurrent bleeding risk while bridging toward full anticoagulation, re-initiation often begins with a reduced dose or with low-molecular-weight heparin bridging. This calibrated approach aims to protect patients from rebound thromboembolic events—a risk that can be as lethal as the hemorrhage itself (122).

## 5 Conclusion and future perspectives

The andexanet alfa experience marks a watershed in anticoagulation reversal. Its trajectory—from a landmark in precision pharmacology to a withdrawn product owing to an unacceptable thrombotic risk—has fundamentally recalibrated expectations. The lesson is unequivocal: molecular targeting alone does not confer clinical safety, and surrogate laboratory endpoints such as anti-Xa activity reduction are no substitute for patient-centered outcomes, including mortality and long-term functional recovery.

In the post-andexanet era, the clinical foundation of anticoagulant-associated major bleeding management rests on two agents: idarucizumab for dabigatran reversal and 4F-PCC for factor Xa inhibitor- and warfarin-associated hemorrhage, supported by adjunctive tranexamic acid and, in specific circumstances, protamine sulfate. These agents—accessible, familiar, and supported by decades of real-world experience—remain the practical cornerstones of emergency hemostatic care across most clinical settings globally.

Looking forward, the field is pivoting toward solutions that address the persistent shortcomings of current therapies. Broad-spectrum small molecules such as ciraparantag, if validated in ongoing Phase III trials, may simplify the management of unknown or mixed anticoagulant exposure. Programmable nucleic acid aptamer systems promise a degree of reversibility and control that current agents cannot provide, particularly in short-term, high-intensity anticoagulation scenarios. The parallel development of companion antidotes alongside novel anticoagulants—epitomized by the factor XI inhibitor pipeline—reflects a regulatory and clinical paradigm in which safety infrastructure is no longer an afterthought.

Translating these pharmacological advances into patient benefit will require parallel investment in the systems that surround drug administration. Point-of-care viscoelastic testing, integrated into clinical decision support frameworks, offers a pathway from empirical dosing toward genuinely individualized hemostatic resuscitation. Multidisciplinary “Code Bleed” teams provide the organizational vehicle to deliver this precision at the bedside, particularly for the most critically ill patients.

Ultimately, optimal care of the bleeding anticoagulated patient is not achieved through rigid algorithmic adherence to any single agent or protocol. It demands the continual, judicious balancing of hemorrhagic and thrombotic risks—calibrated to the patient's evolving physiology, the available evidence, and the resources at hand. That equipoise, rather than any individual drug, constitutes the enduring core of clinical wisdom in anticoagulation management.
